# Post-traumatic pulmonary pseudocyst with hemopneumothorax following blunt chest trauma: a case report

**DOI:** 10.1186/1752-1947-6-356

**Published:** 2012-10-19

**Authors:** Dimitris Fagkrezos, Maria Giannila, Petros Maniatis, John Papailiou, Charikleia Triantopoulou

**Affiliations:** 1 Computed Tomography Department, Konstantopouleio General Hospital, Agias Olgas 3-5, Nea Ionia, 14233, Greece; 2 Department of Radiology, Konstantopouleio General Hospital, Agias Olgas 3-5, Nea Ionia, 14233, Greece

**Keywords:** Traumatic pulmonary pseudocyst, lung cyst, blunt chest trauma, pulmonary contusion

## Abstract

**Introduction:**

Post-traumatic pulmonary pseudocyst is an uncommon cavitary lesion of the lung and develops after blunt chest trauma and even more rarely following penetrating injuries. It is generally seen in young adults presenting with cough, chest pain, hemoptysis, and dyspnea. Post-traumatic pulmonary pseudocyst should be included in the differential diagnosis of cavitary pulmonary lesions. We describe the case of a 60-year-old Caucasian Greek woman who sustained traumatic pulmonary pseudocyst with hemopneumothorax due to a blunt chest trauma after a traffic accident.

**Case presentation:**

After a traffic accident, a 60-year-old Caucasian Greek woman sustained a hemopneumothorax due to a blunt chest trauma. There was evidence of an extensive contusion in the posterior and lateral segments of the right lower lobe, a finding that was attributed to an early sign of a cavitation, and the presence of a thin-walled air cavity was detected on the anterior segment of the right lower lobe in the control computed tomography taken 24 hours after admission. Our patient was treated by catheter aspiration, and the findings of computed tomography evaluation about one month later showed complete resolution of one of the two air-filled cavitary lesions. The second pseudocyst also disappeared completely, as shown by the control computed tomography scan performed six months later.

**Conclusions:**

Traumatic pulmonary pseudocyst is a rare complication of blunt chest trauma, and computed tomography is a more valuable imaging technique than chest radiograph for early diagnosis.

## Introduction

Post-traumatic pulmonary pseudocyst is an uncommon complication after blunt or penetrating chest trauma. Blunt chest trauma causes pulmonary contusions, hematomas, or effusions but rarely leads to the formation of a cystic lesion. Post-traumatic pulmonary pseudocyst should be included in the differential diagnosis of cavitary pulmonary lesions. Young adults and children are most commonly affected. This rare lesion – named “traumatic pulmonary pseudocyst” (TPP) – is an infrequently talked about entity, especially among surgeons. Chest computed tomography (CT) is important for early diagnosis.

## Case presentation

**Figure 1:**
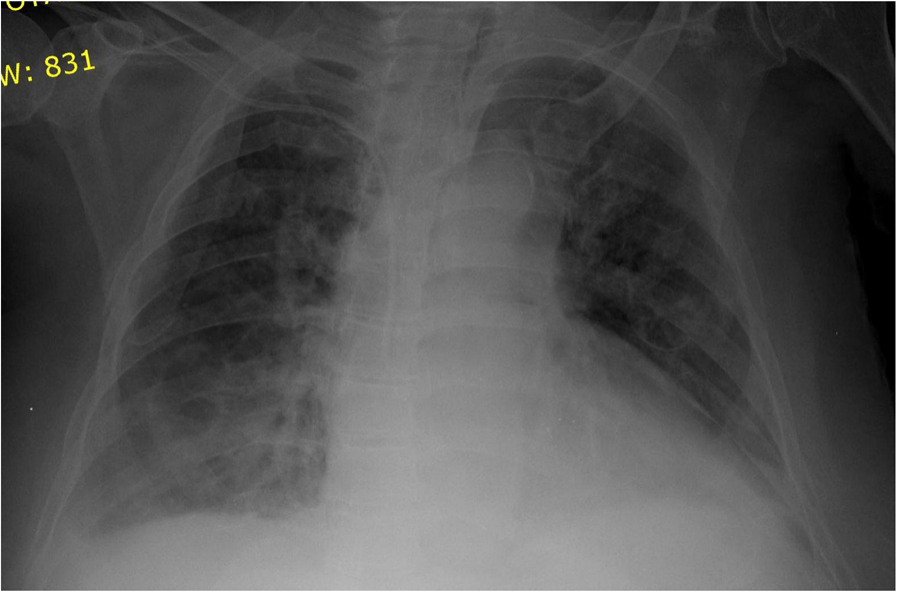
Chest radiograph shows two fractures of fifth and sixth ribs on the right and a cavitary lesion with an air-fluid level in the basal segment of the right lower lobe.

We report the case of a 60-year-old Caucasian Greek woman who sustained TPP with hemopneumothorax due to a blunt chest trauma after a traffic accident. She was a nonsmoker and had a history of diabetes mellitus type II, coronary disease, and heart failure class III. On physical examination, she was hemodynamically stable and well perfused. Auscultation of the lungs revealed decreased respiratory sounds over her right hemithorax, and painful right shoulder motions were noted. Her white blood cell count was 15.6×10^3^/μL, and there were mild increases in serum transaminase, creatine phosphokinase, and lactic dehydrogenase activity. The chest X-rays were consistent with bilateral parenchymal contusion and showed fractures of the fifth and sixth ribs on the right and also showed a cavitary lesion with an air-fluid level in the basal segment of the right lower lobe (Figure 1).

**Figure 2:**
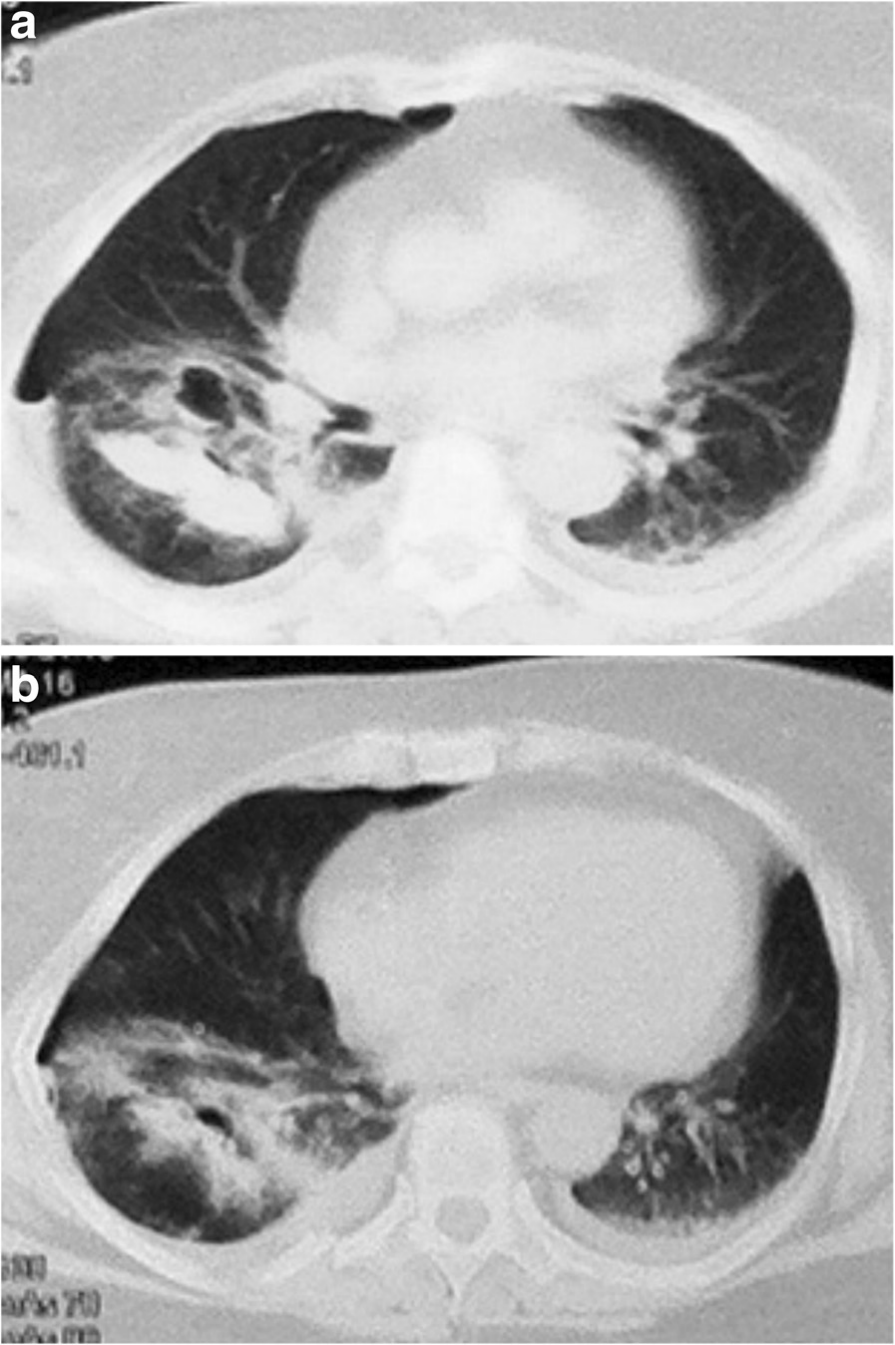
**Axial computed tomography of patient’s blunt chest trauma. **(**A**) Axial computed tomography shows a low-percentage pneumothorax and a thin-walled air cavity on the anterior segment of the right lower lobe in close contact with the interlobar fissure. (**B**) Axial computed tomography shows an extensive contusion in the posterior and lateral segments of the right lower lobe, and the presence of air is demonstrated.

Our patient was admitted to the surgical ward. In the control CT taken 24 hours after admission, a low-percentage pneumothorax and a thin-walled air cavity were detected on the anterior segment of the right lower lobe in close contact with the interlobar fissure (Figure 2a). Also, there was evidence of an extensive contusion in the posterior and lateral segments of the right lower lobe, and the presence of air was demonstrated. This finding was attributed to an early sign of a second cavitation (Figure 2b). During the period between the first and second CT scans, the control was performed with the use of conventional chest X-rays, as it was ordered by the clinicians.

**Figure 3:**
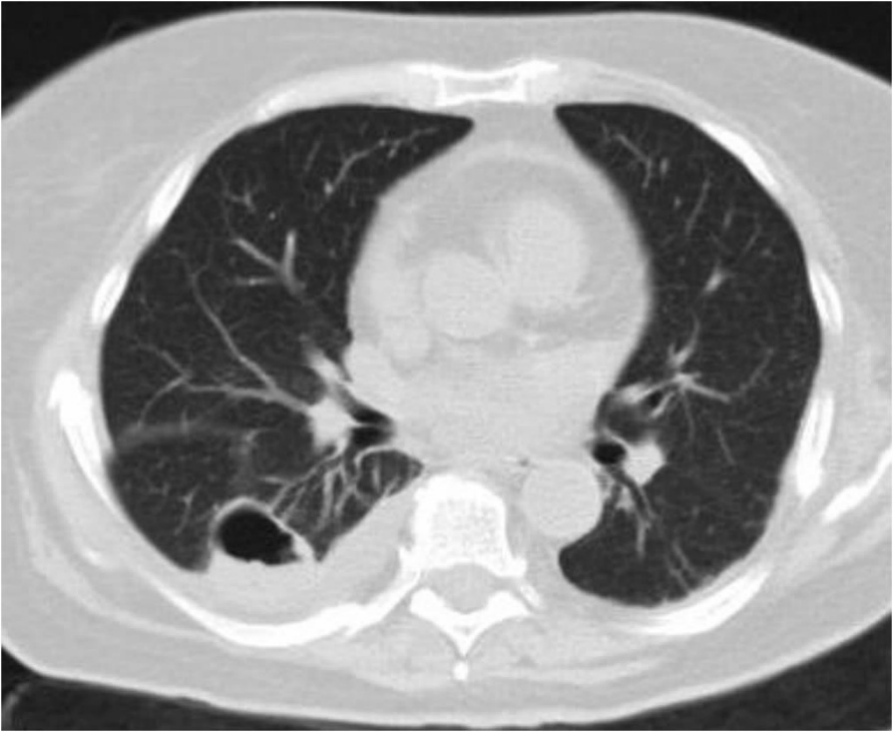
**A computed tomography evaluation about one month after admission showed complete resolution of the air-filled cavitary lesion anteriorly.** In the area of the contusion, a large thin-walled air cavity displaying an air-fluid level is evident.

Pneumothorax was treated, and the findings of a CT evaluation about one month later showed complete resolution of the air-filled cavitary lesion anteriorly, while in the area of the contusion a large thin-walled air cavity displaying an air-fluid level was evident. These findings were consistent with TPP (Figure 3). Additional findings were right pleural effusion and pericardial effusion, which were attributed to heart failure. Our patient was treated conservatively with antibiotherapy. She was asymptomatic thereafter, and the second TPP was completely resolved six months later, as was proven by a follow-up CT scan.

## Discussion

TPPs are most often seen in children and young adults, in whom the thorax is elastic, the visceral pleura intact, and the parenchyma easily injured. Around 85% of cases reported in the literature involved patients below the age of 30 years. Because they are involved more often in motor vehicle accidents and falls, men are affected more than women. In our case report, the patient was 60 years old, which is far from the mean age of patients with TPP. Unlike pulmonary contusions or hematomas, TPPs are rare, developing in less than 3% of patients with pulmonary parenchymal injuries [1].

Santos and Mahendra [2] proposed the term “pseudocyst” because the cyst lacks an epithelial lining. The significance and behavior of the TPP depend on the impact velocity, the degree of chest wall displacement, and the elasticity of the chest wall in blunt chest trauma [3]. It is believed that the more elastic and pliable chest wall of young people and children permits the transmission of kinetic energy more efficiently to the underlying lung parenchyma. The pseudocyst develops via a mechanism that allows the transmission of high compressive force to the lung parenchyma. The concussive forces of a high-velocity impact with low displacement of the chest wall result in a peripheral pseudocyst, whereas the compressive forces of a low-velocity impact with high displacement of the chest wall result in a central pseudocyst [3,4]. An intraparenchymal pulmonary laceration with airway disruption and leaking of air into the pulmonary parenchyma occurs in both mechanisms. The mechanism of TPP due to penetrating injury is not clearly described and requires further investigation. It may develop when air, as a result of “one way” or “check valve” mechanism, is able to enter lacerated parenchyma but unable to escape the pleural space.

The action of rapid compression and decompression lacerates alveoli and interstitium, and the concomitant retraction of the surrounding elastic lung tissue leaves small cavities filled with air or fluid or both, which tend to grow until a pressure balance is achieved between the cavity and the surrounding tissue. Another proposed mechanism is that if the glottis is closed or a bronchus is obstructed at the moment of injury, the air in the compressed lung segment fails to exit fast enough and the parenchyma or interstitium lacerates in a “bursting” pattern and forms a cavity. Patients present with hemoptysis, chest pain, and cough, symptoms that are attributable to the pulmonary parenchymal injury but not to the TPP itself [5]. However, TPP may also be asymptomatic [3].

TPPs are often missed by conventional X-rays, especially if they are obtained when the patient is in the supine position and if the lesion is smaller than 2cm. The diagnostic accuracy of chest radiographies ranges from 24% to 50% of the reported cases [6]. Post-traumatic pulmonary pseudocysts may be identifiable on chest radiograph, but CT is superior for detecting them. Thoracic CT scan can more precisely define the location and size of the cyst and provide early detection and differential diagnosis. However, a series of chest X-rays taken over several days can be useful to differentiate TPP from other kinds of cystic or cavitary lesions, especially if the clinical history of trauma, as in our case, reveals any contusion.

The majority of TPPs are found on lower lobes. TPPs can be single or multiple, unilateral or bilateral. Those of more than 4cm in diameter are usually seen in patients who have multiple injuries with bilateral lesions, whereas those of less than 4cm are usually unilateral [6].

In our patient, in early CT on the second day of admission, there was a right-lung cavitary lesion that completely resolved on evaluation CT one month later and a strong suspicion for a second cavitary lesion in the posterior and lateral segments of the right lower lobe which completely resolved six months later. Post-traumatic pulmonary pseudocysts usually resolve spontaneously but can be complicated and can require surgery. The indications for diagnostic and therapeutic bronchoscopy include endobronchial bleeding, thick sputum, large air leak, mediastinal emphysema, and lobar collapse [1,7,8]. Urgent thoracotomy and lobectomy may be required in the case of massive hemoptysis, which is usually not life-threatening [7].

The indications for video-assisted thoracoscopic surgery or open surgery include prolonged persistence of an air leak, hemothorax due to pseudocyst rupture, failure of lung expansion, progressive enlargement of the pseudocyst, and compression of functional parenchyma. Actually, TPPs may enlarge with positive-pressure ventilation leading to hypoxemia and respiratory deterioration because of inadequate ventilation. In such patients, video-assisted thoracoscopic surgery or open thoracotomy with tube decompression is necessary [1,7].

In case of secondary infection and septic course, antibiotic treatment according to sputum culture antibiograms is the first step. The approach to an infected pseudocyst is similar to that for a lung abscess. If an infected pseudocyst is larger than 2cm or there are unremitting signs of sepsis after 72 hours of antibiotics, the pseudocyst should be percutaneously drained [1]. The indications for surgery are failed conservative treatment, an increase in the size of the pseudocyst, development of complications such as respiratory deterioration, or failure of the pseudocyst to become progressively smaller.

If there is no clinical improvement, early CT-guided catheter drainage should be considered. If, despite the drainage, there is no observed clinical improvement, clinicians could proceed with thoracotomy or thoracoscopy. The average spontaneous time for radiological resolution of TPP is three months.

Cavitary lesions such as cavitating hematomas, lung lacerations, and traumatic pseudocysts detected in patients presenting with trauma may also have a non-trauma-related etiology such as blebs, bullae, congenital cysts, coccidioidomycosis, tuberculosis, hydatid disease, and pneumonia. Particularly in countries where causes of cavitation are endemic, other possible causes should be kept in mind as part of the differential diagnosis. However, clinical or radiological diagnosis of TPP is not difficult. The size, shape, and nature of the wall of the TPP, unlike those of other kinds of cystic or cavitary lesions, change in a relatively short time. Thus, a series of chest X-rays taken over several days can be useful to differentiate TPP from other kinds of lesions, and no extensive examination is necessary [9]. The history of trauma usually delineates any contusion, but if the cavitary lesion in question does not decrease with time, other etiologies must be considered [10,11].

## Conclusions

TPP is a rare complication of blunt chest trauma. Initially, differential diagnosis between pulmonary pseudocyst and pre-existing pulmonary lesions such as congenital pulmonary cysts, post-pneumonia pneumatocele, tuberculosis cavity, abscess, mycotic cavity, or carcinomas may be difficult. CT is more valuable than chest radiograph for early diagnosis. Clinicians should conduct follow-up radiographs or CT scans until the pseudocyst resolves. Prophylactic antibiotics are usually unnecessary. Conservative treatment is an effective way to manage TPP. However, in rare complicated cases, appropriate surgical intervention may be required.

## Consent

Written informed consent was obtained from the patient for publication of this case report and any accompanying images. A copy of the written consent is available for review by the Editor-in-Chief of this journal.

## Abbreviations

CT: computed tomography; TPP: traumatic pulmonary pseudocyst.

## Competing interests

The authors declare that they have no competing interests.

## Authors’ contributions

DF examined the patient, interpreted the findings, and was a major contributor in writing the manuscript. PM and MG analyzed and interpreted the radiologic examination findings and were major contributors in writing the manuscript. CT and JP designed and reviewed the manuscript. All authors read and approved the final manuscript.
